# In Vivo Models for Cholangiocarcinoma—What Can We Learn for Human Disease?

**DOI:** 10.3390/ijms21144993

**Published:** 2020-07-15

**Authors:** Raphael Mohr, Burcin Özdirik, Jana Knorr, Alexander Wree, Münevver Demir, Frank Tacke, Christoph Roderburg

**Affiliations:** Department of Hepatology & Gastroenterology, Campus Virchow Klinikum and Campus Charité Mitte, Charité University Medicine Berlin, 13353 Berlin, Germany; raphael.mohr@charite.de (R.M.); burcin.oezdirik@charite.de (B.Ö.); jana.knorr@charite.de (J.K.); alexander.wree@charite.de (A.W.); muenevver.demir@charite.de (M.D.); frank.tacke@charite.de (F.T.)

**Keywords:** cholangiocarcinoma, animal models, pathophysiology

## Abstract

Cholangiocarcinoma (CCA) comprises a heterogeneous group of primary liver tumors. They emerge from different hepatic (progenitor) cell populations, typically via sporadic mutations. Chronic biliary inflammation, as seen in primary sclerosing cholangitis (PSC), may trigger CCA development. Although several efforts were made in the last decade to better understand the complex processes of biliary carcinogenesis, it was only recently that new therapeutic advances have been achieved. Animal models are a crucial bridge between in vitro findings on molecular or genetic alterations, pathophysiological understanding, and new therapeutic strategies for the clinic. Nevertheless, it is inherently difficult to recapitulate simultaneously the stromal microenvironment (e.g., immune-competent cells, cholestasis, inflammation, PSC-like changes, fibrosis) and the tumor biology (e.g., mutational burden, local growth, and metastatic spread) in an animal model, so that it would reflect the full clinical reality of CCA. In this review, we highlight available data on animal models for CCA. We discuss if and how these models reflect human disease and whether they can serve as a tool for understanding the pathogenesis, or for predicting a treatment response in patients. In addition, open issues for future developments will be discussed.

## 1. Introduction

Cholangiocarcinoma (CCA) is an aggressive cancer of the intra- and extrahepatic bile ducts. With an incidence of 2–3/100.000 in the western world, CCA is a rare disease and accounts for approximately 3% of all gastrointestinal malignancies. However, in countries with a high prevalence of liver fluke infections (e.g., Thailand), CCA represents up to 80% of primary liver tumors [1]. While intrahepatic cholangiocarcinoma (iCCA) shows an increasing incidence in western countries, the incidence of gallbladder carcinoma is declining [2,3,4].

Most cases of CCA are sporadic. Conditions that promote chronic biliary inflammation boost the risk of carcinogenesis (e.g., primary sclerosing cholangitis, liver fluke infections, chronic hepatitis infections, bile duct cysts, hepatolithiasis). CCA is a histologically diverse entity. Animal models and lineage tracing studies indicate that multiple cell types, including hepatic progenitor cells, hepatocytes, cholangiocytes, and peribiliary glands can transform and develop into CCA [1].

The prognosis of patients with advanced-stage CCA is poor. Surgery is the only curative treatment option, but tumors are characterized by high recurrence and frequently by late-stage presentation, where surgery is debarred. Low responsiveness to chemotherapy regimens challenges systemic therapy. Rates of mortality remain high, and overall 5-year survival does not exceed 25–40% [5]. Most recently, promising results from targeted therapies and checkpoint inhibition have opened new pharmacologic treatment options [6,7,8,9].

Animal models represent an important tool to gain better understanding of the molecular pathogenesis of CCA. The establishment of xenograft models and genetically modified organisms resulted in new insights for numerous malignant diseases [10]. However, this approach appears somewhat limited for CCA, as xenografts are usually implanted into immune-compromised hosts and genetical modification derogates the mutational burden. Immune activation, inflammation, and genetic heterogenicity are important players in the development of CCA [1]. 

In this review, we summarize available data on mouse models for CCA. We will briefly recapitulate the current treatment algorithms, discuss how the rodent models reflect tumor biology of human disease, and whether these models could serve as a tool for disease modeling and pharmacosensitivity assays, allowing prediction of treatment responses in humans.

## 2. Current and Emerging Therapeutic Options for CCA

The only curative treatment option for CCA is radical surgery. Liver transplantation is not yet standard of care in CCA [11]. However, disease recurs in up to 70% within 26 months after curative-intended surgery [5]. This high rate of recurrence contributes to a poor overall prognosis, with a median overall survival (OS) of 40 months after liver resection [12,13]. As distant metastasis are more frequently observed than local tumor relapse, adjuvant treatment might be beneficial, especially in high-risk groups, such as patients with positive resection margins or nodal-positive patients [14].

Three recently published phase III studies explored the role of adjuvant chemotherapy compared to surveillance alone after curative surgery [15,16,17]. The BILCAP study demonstrated a significant benefit of capecitabine in this setting, with a median OS of 51.1 vs. 36.4 months (adjusted HR 0.75 (95% CI 0.58–0.97); *p* = 0.028). Nevertheless, subgroup analysis revealed that patients with hilar CCA or tumor stage III did not benefit from adjuvant capecitabine, and both groups showed same disease recurrence after 24 months. The PRODIGE study failed to demonstrate a benefit for adjuvant gemcitabine/oxaliplatin (GEMOX). Interestingly, in case of tumor recurrence, patients that received adjuvant GEMOX showed a trend towards worse median post-relapse OS when compared to those without adjuvant therapy (8.0 vs. 15.2 months; HR 1.55 (95% CI 0.98–2.47); *p* = 0.06). In the BCAT study, gemcitabine did not show a beneficial effect in the adjuvant setting. Additionally, when stratifying according to resection margins or nodal positivity, no significant benefit could be observed. Although these discrepant findings challenged straightforward conclusions regarding adjuvant treatment, current practice has been changed and adjuvant capecitabine is considered standard of care [18].

At diagnosis, most patients already present with a locally advanced or metastatic stage. Thus, a considerable number of patients are only eligible for palliative therapies. The pivotal phase III trials, ABC-02 [19] and BT22 [20], established the current first-line systemic chemotherapy standard with gemcitabine plus a platin derivate. A meta-analysis summarizing both study populations showed the significant superiority of gemcitabine/cisplatin compared to gemcitabine alone in a first-line palliative setting, with a median OS of 11.6 vs. 8.0 months (HR 0.65 (95% CI 0.54–0.78); *p* < 0.001) [21]. Other chemotherapy combinations, mainly with fluorouracil derivates or nab-paclitaxel, are being investigated in ongoing phase II and III trials [22,23]. Promising observations from a phase Ib trial using the gemcitabine pro-drug NUC-1031, designed to overcome tumor mechanisms of drug resistance, could not be supported by efficacy testing using the cytidine deaminase (CDA)-high CCA patient-derived xenograft (PDX) model [24,25].

While adjuvant and palliative therapeutic options were limited to conventional chemotherapy, several molecular mechanisms involved in biliary carcinogenesis were described. These findings opened the door for new personalized therapies in selected patients. The first phase III study in this field confirmed the highly significant effectiveness of ivosidenib in patients with isocitrate dehydrogenase 1 (IDH1) mutations, and promising results for pemigatinib in patients with fibroblast growth factor receptor 2 (FGFR2) fusions were obtained [8,9]. Several oncogenic alterations have already been identified in cholangiocarcinoma cells. Nevertheless, it has to be remembered that CCA are heterogenous tumors, requiring different therapeutic strategies depending on the variable tumor biology. The tumor’s mutational burden and the occurrence of specific genetic alterations (e.g., affecting tyrosine kinase signaling like FGFR, HER2, KRAS, FGFR2 fusions, or the IDH pathway, as well as chromatin-remodeling genes like ARID1A) correlate with the CCA’s anatomic localization within the biliary tract system. Thus, the testing of every patient upfront for genetic alterations might be considered. However, the majority of patients with CCA are negative for biomarkers and new actionable molecular targets are urgently needed. 

## 3. Animal Models

**Figure 1:**
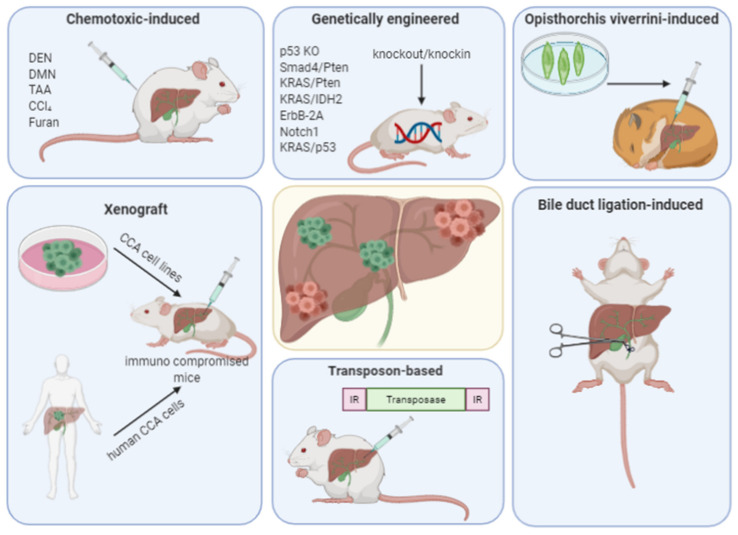
Principles of animal models for cholangiocarcinoma. Some of the different models can be combined. CCA—cholangiocarcinoma, DEN—diethylnitrosamine, DMN—dimethylnitrosamine, TAA—thioacetamide, CCl_4_—carbon tetrachloride, KO—knockout, IDH—isocitrate dehydrogenase, FGFR—fibroblast growth factor receptor.

Animal models of CCA include rodents that develop biliary cancer following exposure to carcinogens, animals with xenograft tumors, or animals with genetic alterations that lead to CCA formation (Figure 1). These models are a crucial bridge between in vitro findings (e.g., characterizing genetic alterations) and pathophysiological understanding, as well as new therapeutic strategies. The ideal animal model of CCA would develop from the biliary tract in an immunocompetent rodent with a functional (and modifiable) microenvironment, recapitulating the biological, molecular, and anatomic characteristics of human disease. Nevertheless, existing models have different limitations. The strengths and weaknesses for the major types of animal models are summarized in Table 1.

## 4. Chemotoxic-Induced Models

Diethylnitrosamine (DEN), dimethylnitrosamine (DMN), furan, thioacetamide (TAA), or carbon tetrachloride (CCl_4_) [26,27,28,29,30,31] have been associated with CCA, inducing genotoxic effects and enhancing tumor formation via the expansion of pre-neoplastic cells. 

### 4.1. Furan Model

The furan-induced model is widely used and regular exposure induces cholangiofibrosis progressing into CCA after 9 to 15 months in rats [31]. Its carcinogenic effect is mainly based on molecular mechanisms of chronic inflammation, such as oxidative deoxyribonucleotide acid (DNA) damage, DNA methylation, and modulation of microribonucleic acids (miRNAs), combined with persistent cell proliferation (e.g., due to loss of connexin 32) [32,33]. The exact genotoxic mechanisms are unclear and controversially discussed. However, some authors described mutations of KRAS and p53 [34,35,36,37,38]. From a molecular point of view, bile ducts display positivity for the hepatocyte growth factor, c-Met, TGF1, and ErbB2, which is similar to the human [39], highlighting the role of this model for examining CCA, though limited to rats [40].

### 4.2. Thioacetamide Model

Oral feeding of rats with thioacetamide (TAA) causes multifocal bile duct proliferation after 9 weeks, followed by microfoci of cancerous cells after 12 weeks, and CCA in all animals after 22 weeks [29,30,41]. TAA is a potent hepatotoxic agent, inducing hepatic fibrosis and cirrhosis upon oxidative bioactivation [42]. Similar to the previous model, the TAA model is also used in almost all cases in rats. Of note, very different strains have been tested, including Lewis, Wistar, Sprague-Dawley, and Zucker fatty rats [29,43]. TAA provokes a severe inflammatory response of the bile tract, coming along with an intense desmoplastic reaction, making this a valuable model to assess cholangio-carcinogenesis in vivo [44]. Although the exact molecular mechanisms leading to CCA are unclear, it seems likely that the bioactivation of TAA produces reactive oxygen species (ROS) and promotes liver toxicity via interference with ribosomal activity [27,42,45]. TAA-induced CCA shares same traits with the human disease. In detail, tumors were immunopositive for different receptor tyrosine kinases, including c-Met and ErbB2, and feature upregulation of the epidermal growth factor, MUC-1, metalloproteinases as well as different estrogen receptors [40]. In summary, this model represents an easy and reproducible toll for CCA research, despite it being standardized only in rats (it is used as a standard hepatic fibrosis model in mice).

### 4.3. Diethylnitrosamine-Left Median Bile Duct Ligation Model

Chemical agents like DEN or DMN are used in combination with cholestatic liver injury models (e.g., left and median bile duct ligations, LMBDL) or liver-flukes infection [46] in order to induce CCA. Whereas DEN is known to cause DNA adducts leading to cancer development, mice receiving either DMN or LMBDL alone do not develop CCA. Induction of chronic cholestasis accelerates progression of CCA, which goes along with the downregulation of miRNA-34a, the upregulation of miRNA-210, and the replacement of Mnt by c-Myc in binding to cyclin D1 [47]. Although this combination model is complex due to the necessity of surgical intervention and long-term feeding, carcinogenesis is relatively fast (28 weeks).

### 4.4. p53 Knockout-Carbon Tetrachloride (CCl_4_) Model

In many types of tumors, the gene coding for p53 is mutated. In some, but not all, tumors, the protein appears to act as a tumor suppressor. p53 plays a role in the regulation of the cell cycle, where it slows down the activity of a number of genes. In human CCA, the TP53 gene mutations occur at a rate of 3–45% [48]. CCl_4_ is a hepatotoxin that is associated with the release of reactive oxygen species [49,50]. Thus, the p53^−^/CCl_4_ model combines the characteristics of a toxic model with those of a genetic model for the induction of cancer. Farazi et al. treated p53^+/+^, p53^+/−^, and p53^−/−^ C57BL/6-mice with CCl_4_ [51]. Invasive CCA occurred in about 55% of p53^−/−^ mice and 20% of p53^+/−^ mice. Metastases represented a rather rare event. On a molecular level, CCA from the p53 knockout CCl_4_ model resemble that of human CCA. Most importantly, activation of c-Met, overexpression of ErbB2, downregulation of E-cadherin, and overexpression of cyclooxygenase (COX)-2 are observed in both human and murine tumors [51]. Important limitations consist of the late tumor development and the relatively low number of mice developing CCA.

### 4.5. Opisthorchis viverrini Model

*Opisthorchis viverrini* (*O. viverrini*) is a trematode parasite that has been associated with CCA development via mechanical damage of the biliary epithelium, as well as immunopathologic reactions to the liver fluke’s antigens. Moreover, liver fluke-induced changes in the biliary tract microbiome are assumed to play an important role in carcinogenesis [52]. In this context, a correlation between high levels of circulating interleukin 6 (IL-6) with the degree of advanced periductal fibrosis in chronic *O. viverrini* infection is described [53,54]. When hamsters were fed with *O. viverrini* followed by oral administration of a subcarcinogenic dose of DMN, 100% of the hamsters developed CCA % [46].

A new monoclonal antibody was generated that specifically detects the so-called S121 antigen in patient tissue and sera, which is highly increased in CCA patients. A time course experiment, in which CCA was induced by a combination of *O. viverrini* and N-nitrosodimethamine in hamsters, suggested that S121 antibody could serve as an early detection marker for CCA [55]. The same hamster model was used to discover upregulation of oxysterol-binding proteins (OSBPs) in *O. viverrini*-induced CCA [56]. OSBP2 and 7 were remarkably expressed in tumor tissue of CCA patients compared to healthy control tissue, which could serve as a future marker for CCA metastasis [57].

## 5. Genetically Engineered Models

Genetically engineered mice (GEM) are an important tool in cancer investigation for analysis of oncogenes, tumor suppressor genes, and complex biological pathways [58,59]. As tumors can arise spontaneously in fully immunocompetent GEM, these models mimic human CCA quite well, particularly CCA subtypes with a specific mutational pattern, and allow the study of stroma-associated responses towards tumor development at early or advanced stages.

These models allow study of the importance of immune modulation, which either induces repair mechanisms or promotes carcinogenesis. The induction of the IL-33/ILC2/IL-13 circuit promotes biliary epithelial repair. However, when stimulated in mice with activated AKT and YAP signaling, two transcriptional coactivators involved in cell proliferation and survival, metastatic CCA is induced [60].

From the model of CCl_4_ administration to p53-knockout mice, we know that p53 deficiency along with chronic bile duct injury and extracellular matrix changes have a major impact on tumor development [51]. In human CCA, different types of p53 mutations were described and their incidence was reported to be 21% [61]. Smad4 is a tumor suppressor gene that was found to be frequently altered in CCA [62], inducing the activation of the proproliferative and antiapoptotic PI3K pathway [63]. Most recently, the identification of IDH1 mutations and FGFR2 fusions resulted in a clinical breakthrough, implementing personalized therapies in these patients [8,9].

### 5.1. Liver-Specific Deletion of Smad4 and Pten

Smad4 and Pten belong to the most frequently mutated tumor suppressor genes in CCA. In 2006, Xu et al. described a murine model for CCA, which is based on the liver-specific deletion of both, Smad4 and Pten, by crossing mice carrying conditional Pten and Smad4 alleles with mice overexpressing a Cre recombinase controlled by an endogenous albumin promoter [64]. Since AlbCre recombines *loxP* sites in precursor cells that might differentiate in both hepatocytes and cholangiocytes, Smad4 and Pten deletions occur in both of these cell types. In these mice, conditional knock-out of Smad4 and Pten is associated with significant hyperplasia of the bile ducts already at 8 weeks of age. These degenerate into dysplasia and finally invasive CCA, occurring with high penetrance at 4–7 months of age. The Smad4/Pten model resembles human CCA on both the histological and molecular level of the tumors. Nevertheless, it is important to note that unlike human CCA, tumors develop in normal livers in the absence of liver injury or inflammation. Moreover, these knock-out mice do not develop metastases, limiting their use as a model for studying advanced CCA of the human.

### 5.2. Liver-Specific Activation of KRAS and Deletion of Pten

Mutations activating the oncogene KRAS are frequent in human CCA [65,66]. Since they are associated with an unfavorable outcome, there is a high clinical need for experimental models allowing this mutation to be specifically addressed. Ikenoue provided a murine model for CCA relying on the simultaneous Alb-Cre-driven activation of mutant KRAS and deletion of Pten [67]. These mice demonstrated an early but stepwise occurrence of intrahepatic malignant lesions that displayed important histological characteristics of CCA. Despite the tumors remaining non-metastatic, mice succumbed to death at a median age of 46 weeks, displaying clinical symptoms frequently observed in human CCA patients, including hemorrhagic ascites, jaundice, and weight loss. Notably, further studies using tamoxifen-regulatable promotors specific for hepatocytes or cholangiocytes revealed that in the described mutant KRAS/Pten mice, CCA originates from biliary cells [67,68]. Similar to the Smad4/Pten model, this model also recapitulates several molecular and histological findings similar to human CCA, but tumors occur in the absence of chronic liver injury and do not metastasize.

### 5.3. Liver-Specific Activation of KRAS and Deletion of Tp53

The tumor suppressor p53, which is encoded by the Tp53 gene, is a phosphoprotein inducing apoptosis or cell cycle arrest when DNA damage occurs. Mutations of p53 have been identified as a frequent event in human CCA. In this context, O’Dell and colleagues generated mice harboring a conditionally activated KRAS mutation and Tp53 deletion to reflect iCCA. Homozygous mutant mice developed tumors already after 9 weeks and died after a median survival time of about 20 weeks. Interestingly, besides CCA, a mixed histological appearance of CCA and hepatocellular carcinoma (HCC) and only HCC developed in about 17% of each case. Within this model, different premalignant lesions (ductular hyperplasia, dysplasia) occur along and frequently adjacent to CCA lesions. A pivotal feature of this model is the very comprehensive formation of metastasis [69].

The specific deletion of adult hepatocytes using an adeno-associated vector under the hepatic-specific thyroid-binding globulin promoter (AAV8-TBG-Cre) revealed that mature hepatocytes do not undergo malignant transformation in the lack of an additional hepatic injury. Strikingly, when these mice were fed with a 3,5-diethoxycarbonyl-1,4-dihydrocollidine (DDC) diet, a feeding model that causes inflammation, ductular reaction, and fibrosis, CCA developed. These findings indicate that hepatocytes are sensitive to KRAS-Tp53-dependent carcinogenesis and can undergo a phenotypic switch to induce CCA development.

### 5.4. Liver-Specific Activation of KRAS and IDH2

Isocitrate dehydrogenase (IDH) is an NADP^+^-dependent metabolic enzyme that catalyzes the oxidative decarboxylation of isocitrate to α-ketoglutarate. Mutant IDH1/2 occurs in about 20% of human CCA, blocking hepatocyte differentiation from progenitor cells [70]. The mutant IDH1 inhibitor ivosidenib (approved for the treatment of AML patients with a susceptible IDH1 mutation) recently demonstrated efficacy in advanced CCA patients with an IDH1 mutation [71]. Saha et al. demonstrated that mice simultaneously bearing a mutant IDH2 and activating KRAS mutation develop CK19-positive liver lesions with a high prevalence [70]. Mice demonstrated peritoneal metastases. Although tumors occurred at a rather later age, this model might be used to study the important subset of IDH-mutated CCA.

### 5.5. ErbB-2A Overexpression

ErbB2 is a receptor tyrosine kinase regulating key tumor cell characteristics, which induces survival, proliferation, and migration [72]. Overexpression of ErbB2 has been described in about 5–7% of human CCA, with much higher rates in gallbladder carcinoma [72]. In line with these data from human, mice overexpressing ArbB2 using the BK5 promoter developed gallbladder carcinoma in about 85% of cases at an extremely young age of 2–3 weeks [73]. At an age of 16 weeks, tumors in other parts of the biliary tract occurred with a lower prevalence. At the molecular level, BK-ErbB-2A demonstrated increased COX-2 levels and an increased activation of the MAPK pathway, which is also observed in human lesions. Thus, BK-ErbB-2A mice represent a model allowing the study of gallbladder adenocarcinoma rather than other types of CCA.

### 5.6. Notch1 Overexpression

Notch is a major regulator of cell development, differentiation, and proliferation. A role for Notch in the embryogenesis of the biliary tree was suggested [74], and aberrations in the Notch pathway were described in many patients with CCA. Especially, Notch 1 and 3 receptors were overexpressed in human CCA tissue. Zender et al. developed a transgenic murine model with constitutive Notch overexpression in albumin-expressing cells (Notch1C:AlbCre) to demonstrate that aberrant Notch activity in hepatic progenitor cells promotes differentiation of these cells towards a biliary lineage, contributing to malignant transformation [75]. Interestingly, both human CCA tissues as well as tumors derived from Notch-overexpressing mice showed significantly increased levels of the cell cycle regulator protein cyclin E. Nonetheless, tumors derived from these models additionally displayed characteristics of HCC or a mixed CCA/HCC phenotype, which together with the high complexity of the model might be seen as an important limitation. Most interestingly, a conversion from differentiated hepatocytes into biliary epithelial cells was observed in mice with overexpression of the Notch pathway. Fan and colleagues demonstrated that iCCA can develop from mature hepatocytes when Notch and AKT signaling are activated [76].

### 5.7. Sleeping Beauty Transposons

As a further development, genetically modified CCA models based on the sleeping beauty (SB) transposon system were described. Transposons represent non-viral vectors for gene delivery. Yamada et al. published a model based on bile duct ligation in C57BL/6 mice that were injected with SB carrying plasmids with active AKT and YAP [77]. Mice developed Sox9^+^/HepPar1^−^ intrahepatic tumors, providing a proof of concept that such models are feasible in mice for the induction of CCA. Similarly, the group of Kühnel and colleagues described an elegant model for the induction of a single CCA lesion in mice [78]. In brief, they injected a transposon plasmid encoding for mutant KRas-G12V into p53-knock-out mice (p53^fl/fl^ mice and co-delivery of a plasmid for Cre-recombinase) directly into the liver. Subsequent electroporation led to CCA development in all animals within 3–5 weeks, demonstrating the oncogenic potential of KRas-G12V on the background of genetic p53 knock-out. If the tumor was left in place, lung metastases occurred, finally leading to the death of the mice. If the tumor was resected, recurrences of the original tumor occurred, both in the form of local recurrences in the liver and in the form of lung metastases in many mice. These led to lethality of the mice, which occurred between 30 and 61 days after resection, depending on the initial tumor size. The model is therefore suited to investigate different clinical scenarios of CCA and to analyze the influence of different molecules by modulating gene expression. The group of Kühnel and colleagues analyzed the efficacy of adjuvant therapy in CCA, providing a proof of concept how the model can be used to directly answer clinical questions in rodent models.

## 6. Implantation Models

### 6.1. Xenograft Models

Xenograft models use human tumor cells or human tissue inserted into immunocompromised mice. Due to the unique condition of the liver (dual vascularization, immunologic microenvironment), orthotopic rather than heterotopic models are considered more suitable to mimic human CCA.

Hudd and colleagues were the first to use an ectopic xenograft model, in which human metastatic CCA cells were subcutaneously injected to immunocompromised mice, leading to CCA formation in 26 of 30 mice [79]. Being reproducible and time effective, xenotransplantation models were frequently used to assess the efficacy of novel therapeutic compounds in vivo. These studies have investigated, for instance, γ-aminobutyric acid (GABA) [80], combinations of salubrinal and rapamycin [81], SC-43 (sorafenib derivate) [82], and the bromodain inhibitor JQ1 [83]. Further, different regulators in signaling pathways, such as SOX17, WTAP, or IL-6, as well as miRNA regulation pathways were described [84,85,86,87,88]. In this context, decreased growth of CCA cells was associated with miRNA-494 upregulation, while an increased tumor growth was observed when miRNA-26a, miRNA-17, miRNA-92, and miRNA-320 were upregulated [89,90,91,92].

Xenograft models may lose genetic heterogeneity due to selection pressure, presence of culture-specific mutations, or gene silencing. A procedure known as patient-derived xenograft (PDX) preserves histopathological and genomic characteristics of a patient’s CCA by transplanting freshly resected tumor pieces. Most recently, it was described that CCA PDXs have highly similar genetic profiles and histology characteristics when compared to primary tumors [93]. This may bring substantial improvements towards a personalized precision medicine and can be used for preclinical testing of antitumor treatment response. Nevertheless, the fact that PDX models can only be used in immunodeficient animals is an important disadvantage, as it prevents the elucidation of the crosstalk between tumor and immune cells.

### 6.2. Syngeneic Models

Syngeneic models have the advantage of inserting rodent CCA cell lines into an animal of the same species with a fully functional immune system. More than a decade ago, Sirica and colleagues injected two rat CCA cell lines directly into the biliary tract of syngeneic Fisher 344 rats. While BDEneu inoculation led, in all animals, to rapid tumor growth with cholestatic disease and peritoneal metastasis, BDEsp injection resulted in a less aggressive non-metastatic iCCA [94]. BDEneu cells, with a mutation of the rat neu oncogene, have similar characteristics compared to human disease, e.g., expression of tumor necrosis factor-related apoptosis-inducing ligand, polo-like kinase 2, and hedgehog pathway activation [94,95]. In this model, CCA develops rapidly and consistently, overcoming the immune and stromal limitations of other xenograft approaches. It mimics a vast desmoplastic reaction characteristic for human disease and has been used to analyze the features responsible for tumor progression [95]. It is frequently used to investigate novel pharmacological therapies. Rizvi and collogues described seven mouse CCA cell lines (SB1–7) that can be injected into mouse livers [96]. Subsequent orthotopic tumors recapitulate histopathologic features of human disease, like desmoplasia, malignant glands, and the expression of CK-19 and SRY box 9. This model enables genetic manipulation of cells prior to insertion and may be utilized to get deeper insights into the tumor–stroma crosstalk and investigate future therapeutic strategies.

## 7. Conclusions

CCAs are a heterogeneous tumor entity, both at the intertumoural and intratumoural levels. They poorly respond to common chemotherapeutic strategies but promise to benefit from personalized therapies. Up to 50% of CCAs have targetable mutations, amplifications, or fusions [97]. This opens new therapeutic opportunities that require intense basic and clinical research.

The thorough molecular characterization of CCA has identified novel molecular subtypes that might explain the different clinical characteristics of CCA, their distinct sensitivities to treatment, and the variable prognosis of patients. Transferring this novel knowledge into animal models might help to assess the relevance of different mutations and genetic alterations for the pathophysiology of human disease. However, only few rodent models were able to be demonstrated to clinically and genetically reflect human disease. Even less models answer the urgent need for preclinical intervention trials. Innovative and molecularly well-defined models of CCA are therefore required to translate novel molecular profiles into diagnostic and therapeutic advances in humans. Immunocompetent rodent cancer models have the advantage over cell-based models (e.g., organoids) to allow characterization of stroma and immune responses as well as immune-oncological therapies, while they may not accurately reflect all mutational patterns observed in human patients. Complementary to animal models, recapitulating CCA in patient-derived culture systems and xenografts could provide new platforms, enabling us to reconstruct the oncogenic steps leading to tumor development. Moreover, such models might help to test the sensitivity of individual tumors to distinct treatments and thereby open the door to personalized therapy for each individual patient.

## Figures and Tables

**Table 1:** Strengths and weaknesses of the main rodent models of cholangiocarcinoma.

Model	Strengths	Weaknesses
**Toxic and Surgery**	Early stage assessment of carcinogenesis inflammatory background	slow tumor development procedures mainly adapted to rats
**Genetically Engineered Mice**	recapitulation of most common genetic alterations early stage assessment of carcinogenesis immune-competent animals allow the study stroma–immune–tumor interactions	expensive/technically challenging no inflammatory background
**Implantation**		
Syngeneic	fully functional immune system preservation of tumor microenvironment	incomplete mimicry of genetic heterogeneity of human CCA
PDX	preservation of histopathologic, transcriptomic, and genomic characteristics of a patient’s CCA testing of chemotherapeutic drug response/personalized precision medicine	absence of tumor microenvironment immunodeficient host

## Abbreviations

CCA	cholangiocarcinoma
CCl_4_	carbon tetrachloride
CDA	cytidine deaminase
CI	confidence interval
COX	cyclooxygenase
DDC	3,5-diethoxycarbonyl-1,4-dihydrocollidine
DEN	diethylnitrosamine
DMN	dimethylnitrosamine
DNA	deoxyribonucleotide acid
FGFR	fibroblast growth factor receptor
GABA	γ-aminobutyric acid
GEM	genetically engineered mice
GEMOX	gemcitabine/oxaliplatin
HCC	hepatocellular carcinoma
HR	hazard ratio
iCCA	intrahepatic cholangiocarcinoma
IDH	isocitrate dehydrogenase
IL	interleukin
LMBDL	left and median bile duct ligations
miRNA	micro ribonuleic acid
MPP	metalloproteinase
*O. viverrini*	*Opisthorchis viverrini*
OS	overall survival
OSBP	oxysterol binding protein
PDX	patient-derived xenograft
Pten	phosphatase and tensin homolog deleted chromosome 10
ROS	reactive oxygen species
SB	sleeping beauty
TAA	thioacetamide
TGF	transforming growth factor
TKI	tyrosine-kinase inhibitors
VEGFR	vascular endothelial growth factor
